# Supplementary material for: scm6A-seq reveals single-cell landscapes of the dynamic m6A during oocyte maturation and early embryonic development
Source: Nat Commun. 2023 Jan 19;14:315. doi: 10.1038/s41467-023-35958-7 (PMC9852475; doi:10.1038/s41467-023-35958-7)
Supplement: Supplementary file 1 — Supplementary information [file 41467_2023_35958_MOESM1_ESM.pdf]

Supplementary Information

scm^{6}A-seq reveals single-cell landscapes of the dynamic m^{6}A during oocyte maturation and early embryonic development

Huan Yao, Chun-Chun Gao, Danru Zhang, Jiawei Xu, Gege Song, Xiu Fan, Dao-Bo Liang, Yu-Sheng Chen, Qian Li, Yanjie Guo, Yu-Ting Cai, Lulu Hu, Yongliang Zhao, Yingpu Sun, Ying Yang, Jianyong Han, Yun-Gui Yang

Supplementary Figure 1

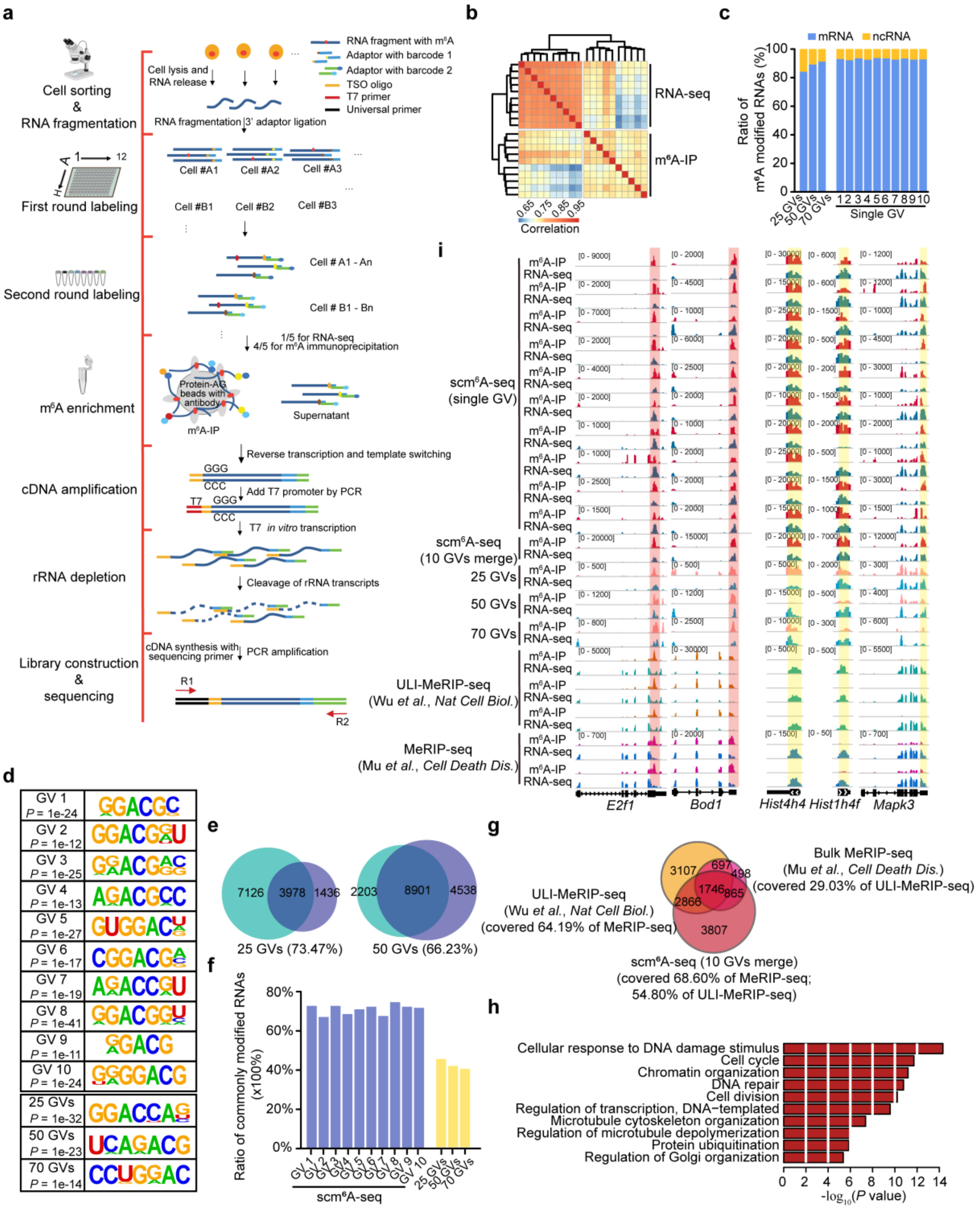

Supplementary Fig. 1 Establishment of scm^{6}A-seq.

(a) Experimental workflow of scm^{6}A-seq. The carton image of stereo microscope is created with BioRender.

(b) Heatmap depicting the high correlations of RNA sequencing (RNA-seq) and m^{6}A immunoprecipitation (IP) data obtained from individual germinal vesicle (GV) oocytes by scm^{6}A-seq.

(c) Bar plot showing the proportion of m^{6}A modified RNAs in individual and bulk GV oocytes.

(d) The m^{6}A motif sequence identified in individual and tens of (25, 50 and 70) GVs as determined by scm^{6}A-seq. The *P* values were calculated using default parameters using hypergeometric test of Homer software.

(e) Venn diagram showing the commonly identified m^{6}A-modified RNAs between 70 GVs and 25 or 50 GVs. The light green pie chart represents the modified RNAs in 70 GVs, blue pie charts show 25 or 50 GV oocytes as labeled.

(f) Bar-plot displaying the ratio of modified RNAs detected in individual GVs to the commonly modified RNAs. Commonly modified RNAs were identified in more than 6 of 10 GVs by scm^{6}A-seq.

(g) Venn diagram displaying the overlap of m^{6}A modified RNAs in GV oocytes by scm^{6}A-seq, ULI-MeRIP-seq (CRA003985) and bulk MeRIP-seq (CRA003041).

(h) Bar-plot showing the enriched Gene Ontology (GO) terms of the conserved modified RNAs (1746) in GV oocytes by scm^{6}A-seq, ULI-MeRIP-seq and bulk MeRIP-seq. The enrichment and *P* value was calculated with default parameters using hypergeometric test of functional annotation in DAVID database.

(i) Genome browser displaying the conserved modified RNAs identified in GV oocytes by scm^{6}A-seq, low-input (ULI-MeRIP-seq) (CRA003985) and bulk MeRIP-seq (CRA003041). The light red and yellow rectangle boxes represent the detected m^{6}A peaks.

Supplementary Figure 2

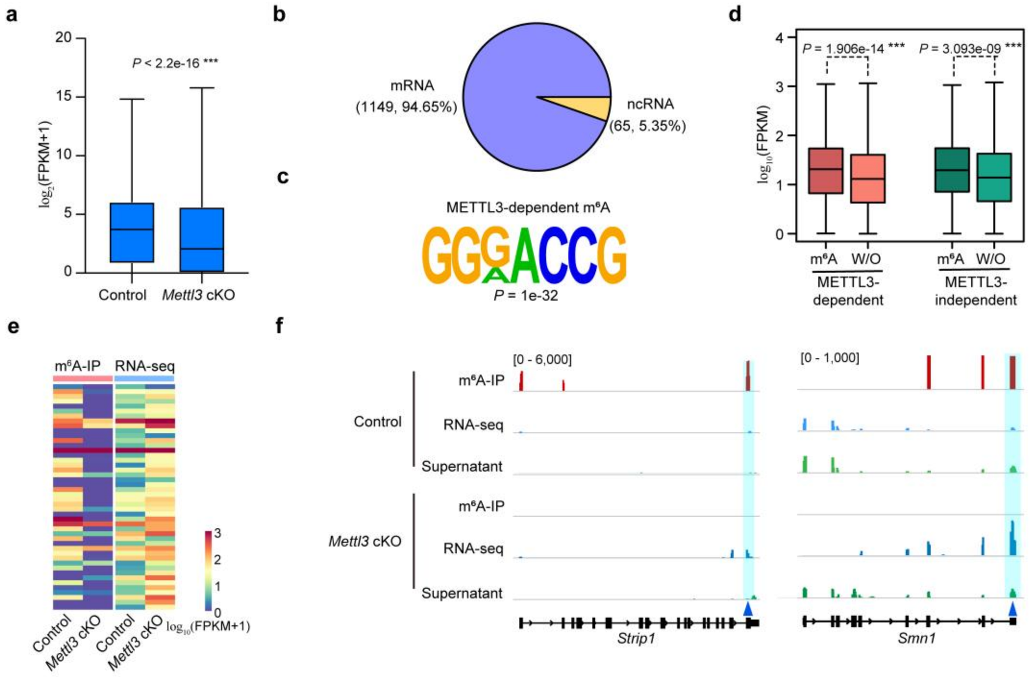

Supplementary Fig. 2 m^{6}A-modified RNAs in germinal vesicle (GV) oocytes are preferentially degraded.

(a) The m^{6}A level decreased significantly in *Mettl3* conditional knockout (cKO) GV oocytes, as proven by unpaired student *t* test of the fragments per kilobase of transcript per million mapped reads (FPKM) values of individual genes (n=4205) calculated using m^{6}A-IP data. The middle lines of the boxes represent the medians of datasets. The upper and bottom lines of the boxes are respectively the upper quantile and the lower quantile of the data. The upper and lower lines of the boxes mark the upper and lower limits of these datasets respectively. Two-sided *P* value were calculated using unpaired student *t* test with $P < 2.2e-16$. ***, $P < 0.001$.

(b) Pie chart displaying the ratio of METTL3-dependent m^{6}A modified RNA types.

(c) The m^{6}A motif of METTL3-dependent peaks. The *P* value was calculated with default parameters using hypergeometric test of Homer software.

(d) Boxplot showing the RNA abundance of m^{6}A modified and unmethylated RNAs in control and *Mettl3* cKO GV oocytes. n (METTL3-independent) = 857, n (METTL3-dependent) =1214. The middle lines of the boxes represent the medians of datasets. The upper and bottom lines of the boxes are respectively the upper quantile and the lower quantile of the data. The upper and lower lines of the boxes mark the upper and lower limits of these datasets respectively. *P* values were determined by one-sided Wilcoxon test, $P = 1.906e-14$ in control oocytes, and $P = 3.093e-09$ in *Mettl3* cKO oocytes. *** $P < 0.001$.

(e) Heatmap displaying the METTL3-dependent-modified RNAs with significantly up-regulated expression levels in scm^{6}A-seq datasets.

(f) Genome browser depicting METTL3-dependent-modified RNAs with up-regulated expression upon *Mettl3* silencing. The yellow rectangle boxes represent the identified m^{6}A peaks in the *Strip1* and *Smn1* transcripts.

Supplementary Figure 3

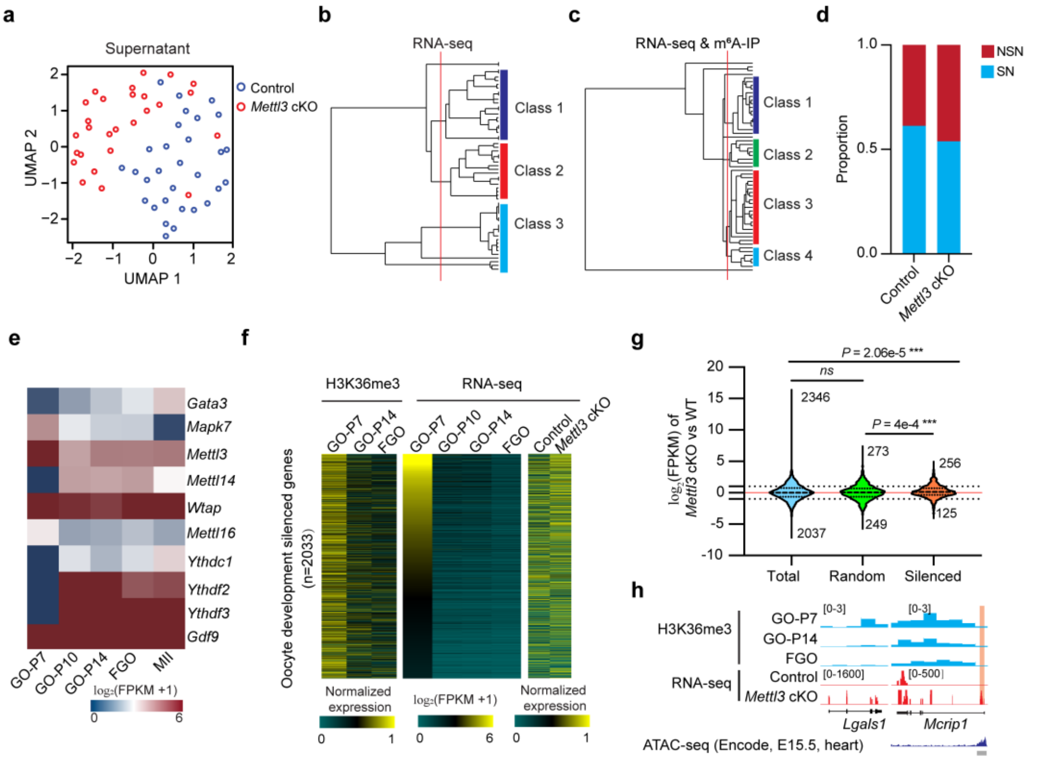

Supplementary Fig. 3 scm^{6}A-seq distinguishes non-surrounded nucleolus (NSN) and surrounded nucleolus (SN) oocytes.

(a) Uniform manifold approximation and projection (UMAP) plot based on the supernatant read count matrix.

(b-c) Dendrogram plot showing the hierarchical clustering results of the analysis of the UMAP distance matrix in 2-dimensional space **(b)** and the results of the combined data of the relative m^{6}A level and UMAP distance matrix in 2-dimensional space **(c)**. The cutoff of height was shown by the red line.

(d) Bar-plot displaying the proportion of SN and NSN oocytes in the control and *Mettl3* cKO groups.

(e) Heatmap showing the expression levels (fragments per kilobase of transcript per million mapped reads [FPKM]) of m^{6}A representative RNAs during oocyte growth.

(f) Heatmap showing H3K36me3 enrichment (reads per kilobase of transcript per million mapped reads [RPKM]), RNA sequencing (RNA-seq; FPKM) for oocyte development silenced genes and the relative expression of these genes in control and *Mettl3* cKO oocytes.

(g) Oocyte developmental genes were significantly more stable in *Mettl3* cKO oocytes than in control oocytes. Violin plot showing the fold change in all RNAs, randomly selected RNAs and the development-silenced RNAs between *Mettl3* cKO oocytes and control oocytes. The two-sided P value was calculated by unpaired student t test. P = 2.06e-5 between total and silenced gene sets. P = 4e-4 between random and silenced gene sets, P = 0.84 between total and random gene sets. ***, $P < 0.001$.

(h) Snapshots showing H3K36me3 in growing oocytes at the postnatal day (P) 7 (GO-P7), GO-P14 and fully grown oocytes (FGOs) for presentative up-regulated genes in *Mettl3* cKO oocytes and RNA-seq in control and *Mettl3* cKO samples for these genes.

Supplementary Figure 4

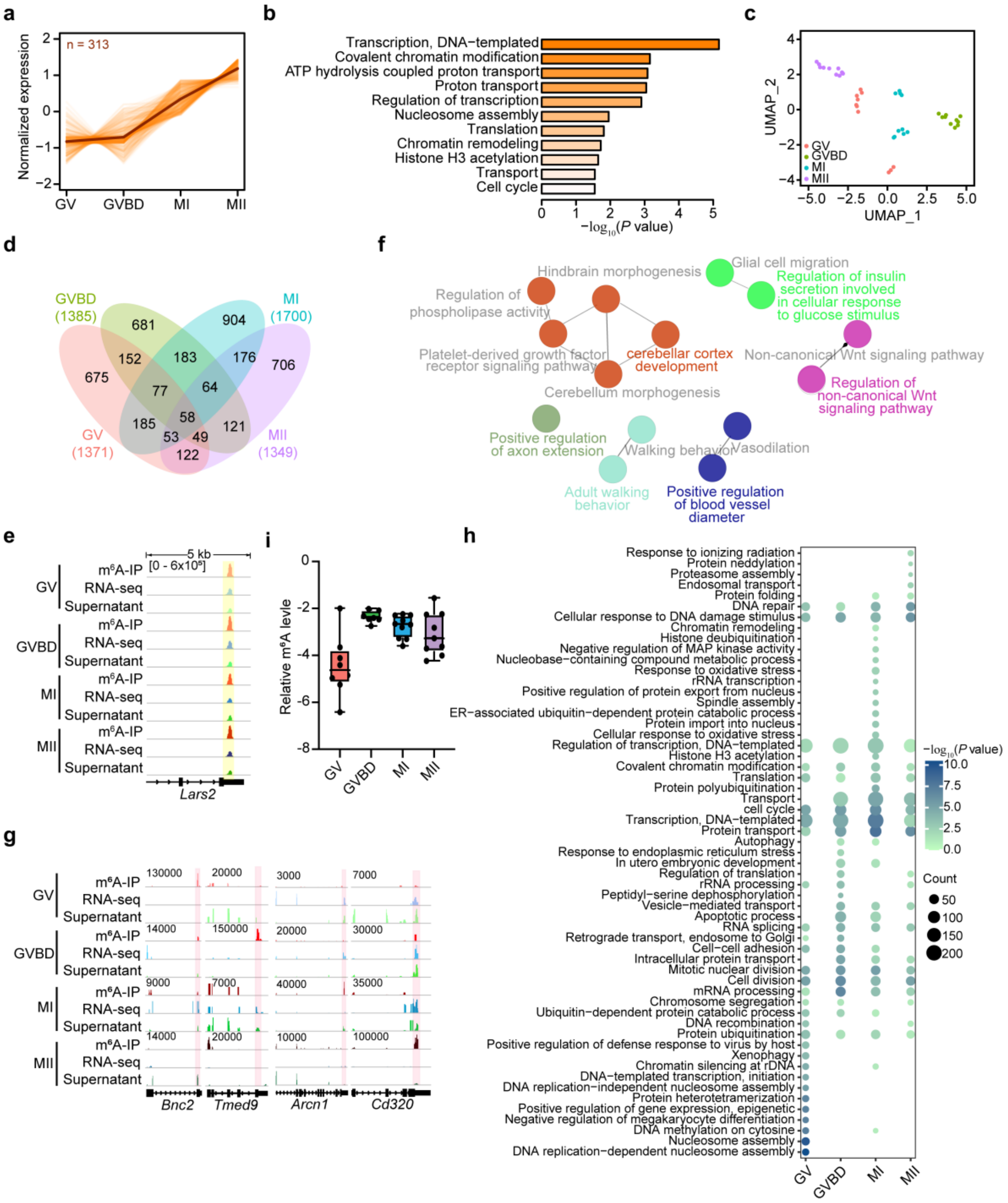

Supplementary Fig. 4 scm^{6}A-seq reveals the dynamic RNA methylome and transcriptome during oocyte maturation.

(a) Line plot showing the increased expression of the gene set during oocyte maturation as determined by K-means clustering.

(b) Bar-plot displaying the enriched Gene Ontology (GO) terms for the gene set presented in **(a)**. The enrichment and *P* value was calculated with default parameters using hypergeometric test of functional annotation in DAVID database.

(c) UMAP clustering of oocytes in different maturation stages on the basis of the RNA expression data obtained by scm^{6}A-seq.

(d) Venn diagram showing m^{6}A modified RNAs in different oocyte maturation stages.

(e) Integrated genomics viewer (IGV) tracks showing the commonly memthylated RNAs during oocyte maturation.

(f) ClueGO analysis of the commonly modified RNAs in different oocyte maturation stages.

(g) Integrated genomics viewer (IGV) tracks showing the stage-specific modified RNAs during oocyte maturation.

(h) Bubble chart showing the enrichment of GO terms for the modified RNAs in different stages of oocyte maturation. Bubble size represents the number of identified modified RNAs enriched in each term, and *P* values from nonsignificance to high significance were shown in colors ranging from light green to blue. The enrichment and *P* value were calculated with default parameters using hypergeometric test of functional annotation in DAVID database.

(i) Boxplot depicting the m^{6}A abundance in individual cells during oocyte maturation. n (of each stage) = 10. The middle lines of the boxes represent the medians of datasets. The upper and bottom lines of the boxes are respectively the upper quantile and the lower quantile of the data. The upper and lower lines of the boxes mark the upper and lower limits of these datasets respectively.

Supplementary Figure 5

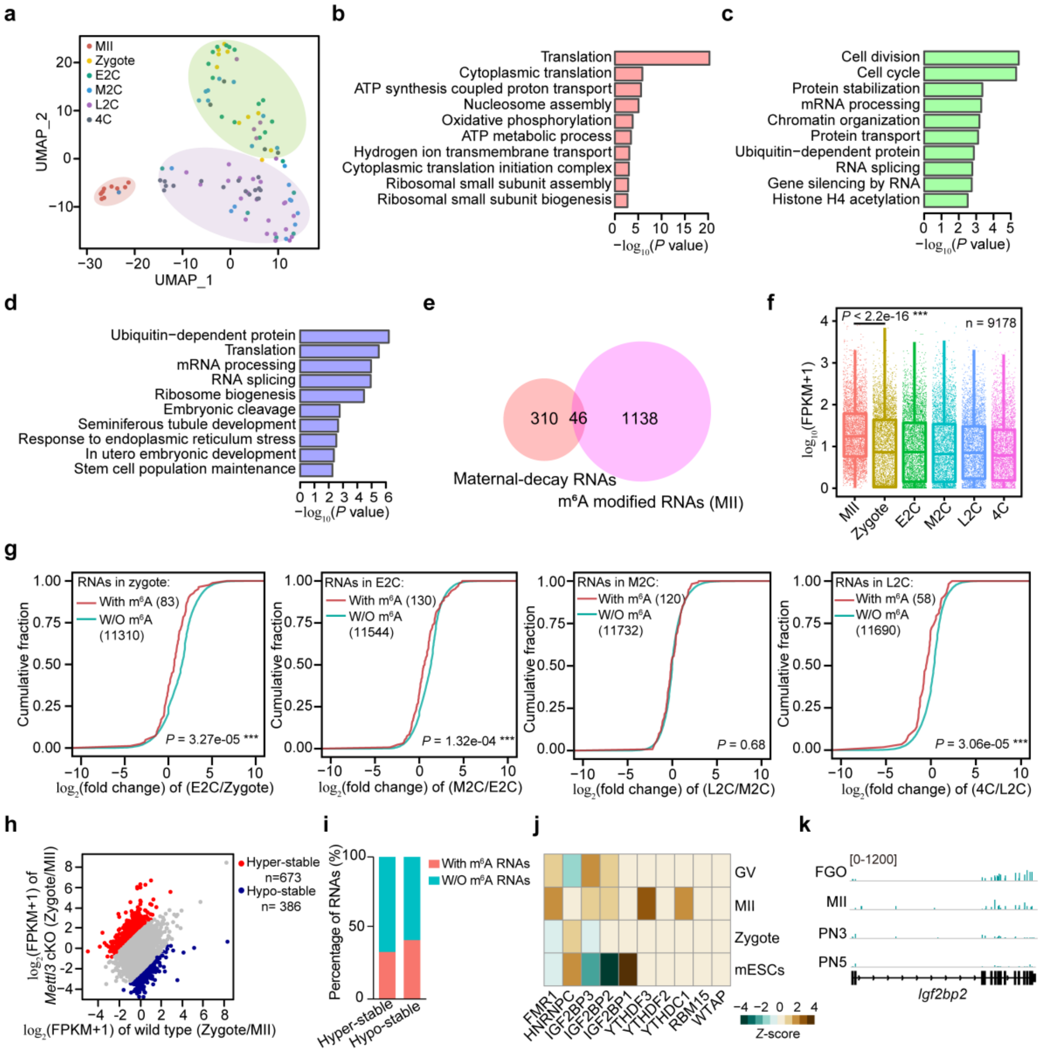

Supplementary Fig. 5 scm^{6}A-seq delineates the dynamics of the RNA methylome and transcriptome during early embryonic development.

(a) UMAP clustering for cells in early embryonic development using RNA expression data obtained by scm^{6}A-seq.

(b-d) Bar plot displaying the enriched GO terms of overlapping maternal-decay RNAs, minor zygotic genome activation (ZGA) RNAs and major ZGA RNAs **(Fig. 5a)**. The enrichment and *P* value were calculated with default parameters using hypergeometric test of functional annotation in DAVID database.

(e) Venn diagram showing the overlap of maternal-decay RNAs with m^{6}A modified RNAs of MII oocytes.

(f) Box and whisker plot showing the expression of unmodified RNAs in oocytes from the MII to the 4-cell embryos. The middle lines of the boxes represent the medians of datasets. The upper and bottom lines of the boxes are respectively the upper quantile and the lower quantile of the data. The upper and lower lines of the boxes mark the upper and lower limits of these datasets respectively. *P* values were determined by one-sided Wilcoxon test, $P < 2.2e-16$. ***, $P < 0.001$ (n= 9178).

(g) Cumulative curves showing the abundance differences of the m^{6}A modified and unmodified RNAs ($\log_2$(fold change)) at two adjacent developmental time points, from the zygote to the 4-cell stage. *P* values were calculated by one-sided Wilcoxon test. *, $P < 0.05$; **, $P < 0.01$; ***, $P < 0.001$.

(h) Scatterplot showing expression changes (zygote versus MII) between the *Mettl3* cKO group (n (MII) = 4, n (zygote) = 4) and control group (n (MII) = 4, n (zygote) = 5). |Fold change| ≥ 4 was used as the cut-off to identify hyper-stable and hypo-stable genes.

(i) Bar plot showing the proportion of m^{6}A modified and unmodified RNAs in hyper-stable and hypo-stable gene sets.

(j) Protein abundance of expressed m^{6}A related proteins in GV oocytes, MII oocytes, zygotes and embryonic stem (ES) cells.

(k) Integrated genomics viewer (IGV) tracks showing the translation signal of *Igf2bp2*.

Supplementary Figure 6

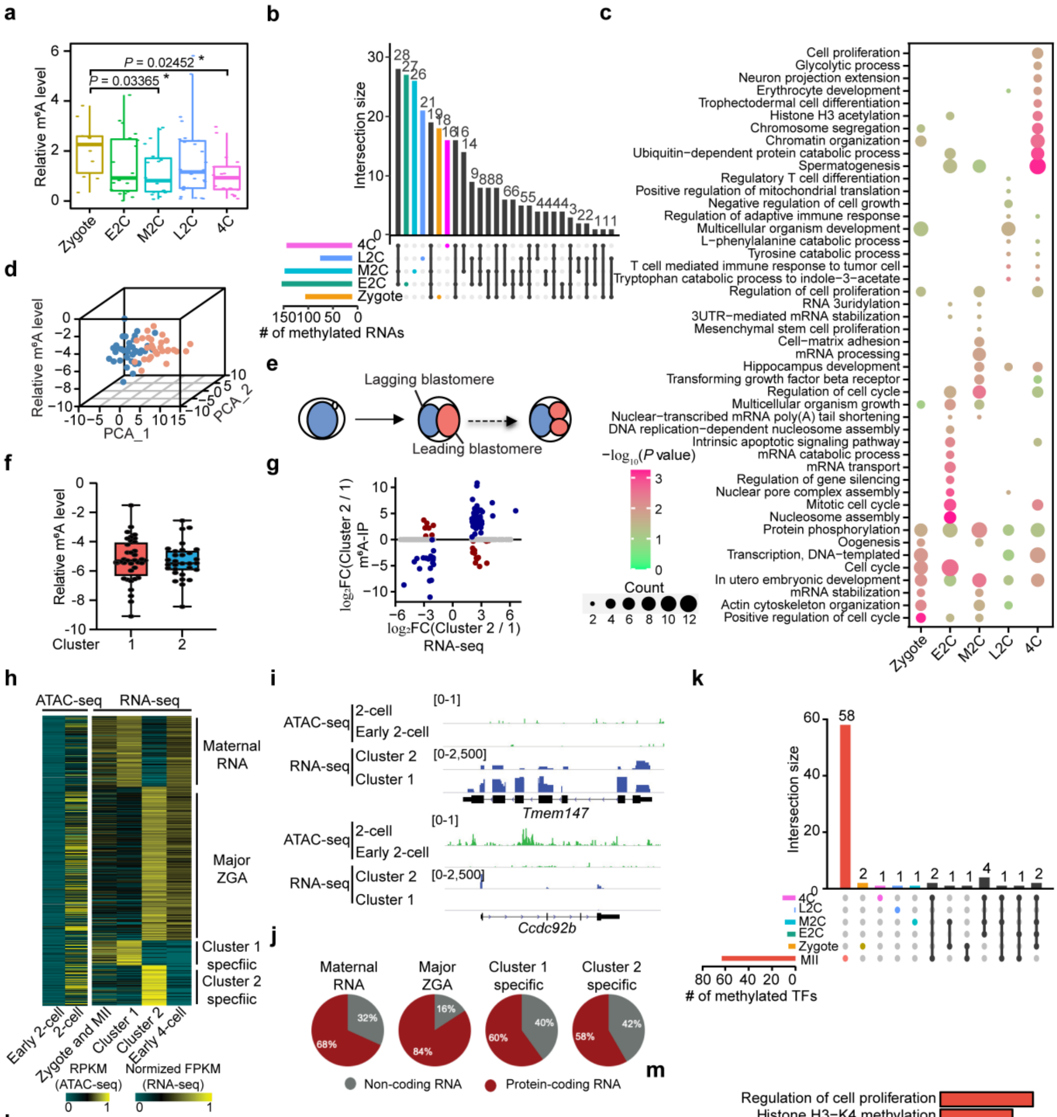

l

Stages	# of RNAs	# of TF	# of methyRNAs	# of methyTF	Hyper.test
MI	5230	207	1349	63	0.071638
Zygote	8226	372	95	6	0.259187
E2C	9393	416	143	7	0.44861
M2C	9017	412	137	9	0.174559
L2C	9797	439	65	1	0.949708
4C	8727	405	133	11	**0.044698***

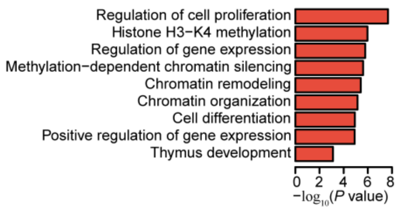

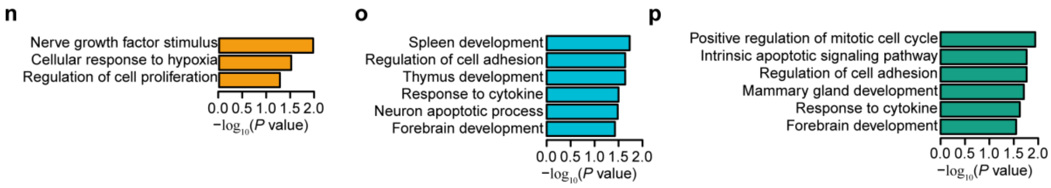

Supplementary Fig. 6 scm^{6}A-seq reveals the roles played by m^{6}A-modified transcription factor (TF) mRNAs in transcription and differentiation processes.

(a) Box and whisker plot displaying the m^{6}A enrichment according to m^{6}A-IP and RNA-seq data obtained from scm^{6}A-seq in individual cells from the MII oocyte to the 4-cell stage. The middle lines of the boxes represent the medians of datasets. The upper and bottom lines of the boxes are respectively the upper quantile and the lower quantile of the data. The upper and lower lines of the boxes mark the upper and lower limits of these datasets respectively. P values were calculated by one-sided Wilcoxon test, P = 0.03365 between zygote and mid-2-cell and P = 0.02452 between zygote and 4-cell, *, $P < 0.05$. n (Zygote) =10, n (Early 2-cell) =20, n (Mid 2-cell) = 20, n (Late 2-cell) = 26, n (4-cell) = 20.

(b) UpsetR plot showing the frequency of the detected m^{6}A modified RNAs during early embryonic development.

(c) Bubble chart of the enriched GO terms for m^{6}A-modified RNAs during early embryonic development.

(d) 3D UMAP clustering showing the classification of cells at the 2-cell stage. The relative m^{6}A level of m^{6}A-IP to RNA-seq as determined by scm^{6}A-seq was used as the third component for clustering.

(e) Schematic diagram of the dys-synchrony of cell development in 2-cell stage embryos.

(f) Relative m^{6}A level of m^{6}A-IP to RNA-seq as determined by scm^{6}A-seq for the cell populations shown in **(d)**. The middle lines of the boxes represent the medians of datasets. The upper and bottom lines of the boxes are respectively the upper quantile and the lower quantile of the data. The upper and lower lines of the boxes mark the upper and lower limits of these datasets respectively. n (Class 1) = 30, n (Class 2) = 36.

(g) Point plot showing RNA abundance difference of m^{6}A-IP and RNA-seq for the cell populations in shown in **(d)**.

(h) Heatmap showing transposase-accessible chromatin with high-throughput sequencing ATAC-seq (RPKM), and RNA-seq (FPKM) data of differentially expressed

genes between Cluster 1 and Cluster 2 blastomeres (fold change > 4). RNAs were clustered into four classifications based on gene expression data.

(i) IGV snapshots showing the tracks of representative differentially expressed genes among both maternal RNA and ZGA genes.

(j) The proportion of protein-coding and non-coding RNAs in the RNA groups shown in **(h)**.

(k) UpsetR plot displaying the frequency of modified TF mRNAs in different embryonic development stages.

(l) The table of the detected RNAs, modified RNAs and modified TF mRNAs in different embryonic development stages. A hypergeometric test was used for statistical analysis.

(m-p) The enriched GO terms for m^6A-modified TF mRNAs in MII oocyte **(m)**, zygote **(n)**, early 2-cell embryo **(o)** and mid-2-cell embryo **(p)**. The enrichment and *P* value were calculated with default parameters using hypergeometric test of functional annotation in DAVID database.
