# Supplementary material for: scm6A-seq reveals single-cell landscapes of the dynamic m6A during oocyte maturation and early embryonic development
Source: Nat Commun. 2023 Jan 19;14:315. doi: 10.1038/s41467-023-35958-7 (PMC9852475; doi:10.1038/s41467-023-35958-7)
Supplement: Supplementary file 3 — Description of Additional Supplementary Files [file 41467_2023_35958_MOESM3_ESM.pdf]

Description of Additional Supplementary Files

File Name: Supplementary Data 1
Description: Identification of m^6A peaks for single GV oocyte as determined by scm^6A-seq, related to Figure 1.

File Name: Supplementary Data 2
Description: m^6A peaks and modified RNAs in tens of GV oocytes, related to Figure 1.

File Name: Supplementary Data 3
Description: The expression of METTL3-dependent m^6A modified RNAs in control and Mettl3 cKO GV oocytes, related to Figure 2.

File Name: Supplementary Data 4
Description: The m^6A altas during oocyte maturation, related Figure 4.

File Name: Supplementary Data 5
Description: Maternal decay and ZGA transcripts identification by DESeq2, related to Figure 5.

File Name: Supplementary Data 6
Description: The expression changes of m^6A-modified RNAs during early embryonic development, related to Figure 5.

File Name: Supplementary Data 7
Description: The whole transcriptome of Mettl3 cKO MII oocytes and zygotes, related Figure 5.

File Name: Supplementary Data 8
Description: The m^6A altas during early embryonic development, related Figure 6.

File Name: Supplementary Data 9
Description: The adapters and primers used in scm^6A-seq.

File Name: Supplementary Data 10
Description: The barcode sequences for single-cell labeling.
