# Supplementary material for: scm6A-seq reveals single-cell landscapes of the dynamic m6A during oocyte maturation and early embryonic development
Source: Nat Commun. 2023 Jan 19;14:315. doi: 10.1038/s41467-023-35958-7 (PMC9852475; doi:10.1038/s41467-023-35958-7)
Supplement: Supplementary file 14 — Reporting Summary [file 41467_2023_35958_MOESM14_ESM.pdf]

nature portfolio

Corresponding author(s): Yun-Gui Yang

Last updated by author(s): Jan 6, 2023

Reporting Summary

Nature Portfolio wishes to improve the reproducibility of the work that we publish. This form provides structure for consistency and transparency in reporting. For further information on Nature Portfolio policies, see our Editorial Policies and the Editorial Policy Checklist.

Statistics

For all statistical analyses, confirm that the following items are present in the figure legend, table legend, main text, or Methods section.

n/a	Confirmed	
☐	☒	The exact sample size (*n*) for each experimental group/condition, given as a discrete number and unit of measurement
☐	☒	A statement on whether measurements were taken from distinct samples or whether the same sample was measured repeatedly
☐	☒	The statistical test(s) used AND whether they are one- or two-sided<br>*Only common tests should be described solely by name; describe more complex techniques in the Methods section.*
☒	☐	A description of all covariates tested
☒	☐	A description of any assumptions or corrections, such as tests of normality and adjustment for multiple comparisons
☒	☐	A full description of the statistical parameters including central tendency (e.g. means) or other basic estimates (e.g. regression coefficient) AND variation (e.g. standard deviation) or associated estimates of uncertainty (e.g. confidence intervals)
☐	☒	For null hypothesis testing, the test statistic (e.g. *F*, *t*, *r*) with confidence intervals, effect sizes, degrees of freedom and *P* value noted<br>*Give P values as exact values whenever suitable.*
☒	☐	For Bayesian analysis, information on the choice of priors and Markov chain Monte Carlo settings
☒	☐	For hierarchical and complex designs, identification of the appropriate level for tests and full reporting of outcomes
☐	☒	Estimates of effect sizes (e.g. Cohen's *d*, Pearson's *r*), indicating how they were calculated

Our web collection on statistics for biologists contains articles on many of the points above.

Software and code

Policy information about availability of computer code

Data collection	Sequencing data were produced by Illumina NovaSeq 6000 platform.
Data analysis	The template switch oligo (TSO) sequence of raw sequencing reads was trimmed by Cutadapt (version 2.10), and then HISAT2 (version 2.0.5) was used for sequencing reads mapping to the Mus musculus (mouse) genome (GRCm38/mm10). Samtools (version 1.6) , featureCounts (version 2.0.3) , BEDTools (version 2.30.0) and DeepTools (version 3.3.1) were applied for data processing. StringTie (version 2.2.1) was used to perform gene expression level analysis. MACS2 (version 2.1.4) was used for m6A peaks calling. HOMER (version 4.11.1) was used to find the m6A motifs. The Go enrichment analysis was performed using Metascape. R packages Monocle 3 and Seurat 4.0 were used for single cell analysis. MEME-Chip (Version 5.5) was used for transcription factors enrichment. Custom manuscript for data processing can be found at https://zenodo.org/record/6809317#.YsfA0IRByUk

For manuscripts utilizing custom algorithms or software that are central to the research but not yet described in published literature, software must be made available to editors and reviewers. We strongly encourage code deposition in a community repository (e.g. GitHub). See the Nature Portfolio guidelines for submitting code & software for further information.

Data

Policy information about availability of data

All manuscripts must include a data availability statement. This statement should provide the following information, where applicable:

- Accession codes, unique identifiers, or web links for publicly available datasets
- A description of any restrictions on data availability
- For clinical datasets or third party data, please ensure that the statement adheres to our policy

The raw sequencing files have been deposited at Genome Sequence Archive of National Genomics Data Center under the accession number CRA006425 (shared URL: https://ngdc.cncb.ac.cn/gsa/browse/CRA006425). Public data used in this paper: ATAC-seq of early 2-cell and late 2-cell embryos (GSE6658); eCLIP-seq of YTHDF proteins in mESCs (GSE151788); H3K36me3 chip-seq of growing oocytes (GSE112835); Ribo-seq of MII oocytes (GSE169632); ULI-MeRIP-seq data (CRA003985); Bulk MeRIP-seq data (CRA003041). All the data supporting the findings of this study are available within the article, and the Supplementary Information. The immunofluorescence staining (IF) data generated in this study have been deposited in the Mendely Data (https://data.mendeley.com/datasets/dhskwr3r5f/draft?a=69360c62-93e5-415a-b5cf-36b51dcfe0f9). Source data are provided with this paper.

Human research participants

Policy information about studies involving human research participants and Sex and Gender in Research.

Reporting on sex and gender	N/A
Population characteristics	N/A
Recruitment	N/A
Ethics oversight	N/A

Note that full information on the approval of the study protocol must also be provided in the manuscript.

Field-specific reporting

Please select the one below that is the best fit for your research. If you are not sure, read the appropriate sections before making your selection.

☒ Life sciences ☐ Behavioural & social sciences ☐ Ecological, evolutionary & environmental sciences

For a reference copy of the document with all sections, see nature.com/documents/nr-reporting-summary-flat.pdf

Life sciences study design

All studies must disclose on these points even when the disclosure is negative.

Sample size	No statistical method was used to predetermine sample size. Sample sizes were based on those used in previous and preliminary studies from our lab which allow for statically valid comparisons. For each stage of oocytes and embryonic development, we collected at least 4 oocytes/embryos for further studies.
Data exclusions	No data were excluded from analyses.
Replication	All data were single cell data from oocytes and early embryos cells by scm6A-seq without replication.
Randomization	For the single cell m6A-seq in oocytes and embryos, mice were randomized prior treatment. Age and sex-matched animals were used for each experiment. Wild type and knock-out mice were littermates if possible.
Blinding	Blinding was not achieved due to requirements for single cell barcoding.

Reporting for specific materials, systems and methods

We require information from authors about some types of materials, experimental systems and methods used in many studies. Here, indicate whether each material, system or method listed is relevant to your study. If you are not sure if a list item applies to your research, read the appropriate section before selecting a response.

Materials & experimental systems

n/a	Involved in the study
☐	☒ Antibodies
☒	☐ Eukaryotic cell lines
☒	☐ Palaeontology and archaeology
☐	☒ Animals and other organisms
☒	☐ Clinical data
☒	☐ Dual use research of concern

Methods

n/a	Involved in the study
☒	☐ ChIP-seq
☒	☐ Flow cytometry
☒	☐ MRI-based neuroimaging

Antibodies

Antibodies used	Anti-METTL3 antibody (Abcam, Cat # ab195352; 1:500), Donkey anti-Rabbit IgG (H+L) Highly Cross-Adsorbed Secondary Antibody, Alexa Fluor™ 488 conugated (Invitrogen, Cat # A21206; 1:500).
Validation	The anti-METTL3 antibody was used in Fig.3 validated in previous report (Mu, H. et al. (2021). Cell Death Dis, 12, 989, PMID: 34689175).

Animals and other research organisms

Policy information about studies involving animals; ARRIVE guidelines recommended for reporting animal research, and Sex and Gender in Research

Laboratory animals	All mice described (WT, Mettl3flox/flox, Gdf9-cre, Mettl3flox/flox;Gdf9-cre) were kept at C57BL/6J genetic background, and housed under specific pathogen-free (SPF) conditions with 12 light/12 dark cycle at 22 °C with 40% humidity. 4-6 weeks female mice were used for oocytes and embryos collection.
Wild animals	Study did not involve wild animals.
Reporting on sex	Female mice were collected for harvesting oocytes and early embryos for maturation and for embryonic development analysis.
Field-collected samples	Study did not involve samples collected in the field.
Ethics oversight	All animal experiments were approved by the Animal Care and Use Committee of China Agricultural University.

Note that full information on the approval of the study protocol must also be provided in the manuscript.
